# Pressure Tuning Studies of Four-Stranded Nucleic Acid Structures

**DOI:** 10.3390/ijms24021803

**Published:** 2023-01-16

**Authors:** László Smeller

**Affiliations:** Department of Biophysics and Radiation Biology, Semmelweis University, Tűzoltó u. 37-47, 1094 Budapest, Hungary; smeller.laszlo@med.semmelweis-univ.hu

**Keywords:** tetraplex, G-quadruplex, i-motif, high pressure, volume change, unfolding volume, Htel, c-MYC, hepatitis B, FRET

## Abstract

Four-stranded folded structures, such as G-quadruplexes and i-motifs in the genome, have attracted a growing interest nowadays since they have been discovered in the telomere and in several oncogene promoter regions. Their biological relevance is undeniable since their existence in living cells has been observed. In vivo they take part in the regulation of gene expression, in vitro they are used in the analytical biochemistry. They are attractive and promising targets for cancer therapy. Pressure studies can reveal specific aspects of the molecular processes. Pressure tuning experiments allow the determination of the volumetric parameters of the folded structures and of the folding–unfolding processes. Here, we review the thermodynamic parameters with a special focus on the volumetric ones, which were determined using pressure tuning spectroscopic experiments on the G-quadruplex and i-motif nucleic acid forms.

## 1. Introduction

Besides the well-known double helical structure, nucleic acids can form a four-stranded structure as well. One of them is the G quadruplex (GQ) or G-tetraplex, formed by guanine-rich sequences [1,2,3]. The complementary strand enriched in cytosine bases is also able to form four-stranded forms, named the i-motif (iM) or i-tetraplex [4]. These noncanonical structures have gained increasing interest due to their appearance at the crucial positions of the nucleic acids.

Although GQ and iM structures were discovered earlier and were characterized in in vitro studies, it was unclear for a long time whether they are really formed in vivo. Their existence in living cells was proven recently, and they can be detected by specific microscopic techniques in the cell [5,6].

These unique structures were found in several places in the human genome. They were identified in the telomeres and in promoters of several oncogenes as well. These findings made them an attractive target for cancer therapy research [7].

Telomeres are known to be shortened during cell division, but they are elongated by the telomerase enzyme in immortal cancer cells. The formation of GQ structures can hinder the telomerase activity. The development of stabilizing ligands was one of the attempts to hinder the immortality of the tumor cells.

The appearance of GQ-forming sequences was also reported in several human gene promoters including in those of c-MYC, VEGF, RET, BCL-2, c-KIT, K-RAS, hTERT, HIF-1α, MtCK, PDGF-Rβ, PDGF-A and c-MYB [8]. Potentially, GQ-forming DNA sequences have been reported in the promoter regions of genes involved in proliferation and growth. They are more abundant in the oncogene genes compared to the tumor suppressor ones [9].

G-quadruplexes are present in viral genomes in both DNA and RNA viruses, and they take part in the key steps of viral infection of the cell from replication to encapsidation [10,11,12,13,14,15]. This research was even further accelerated by the recent pandemic: the focus of the research turned to the genome of the SARS-CoV-2 virus [15,16,17,18,19].

## 2. The G-Quadruplex

**Figure 1:**
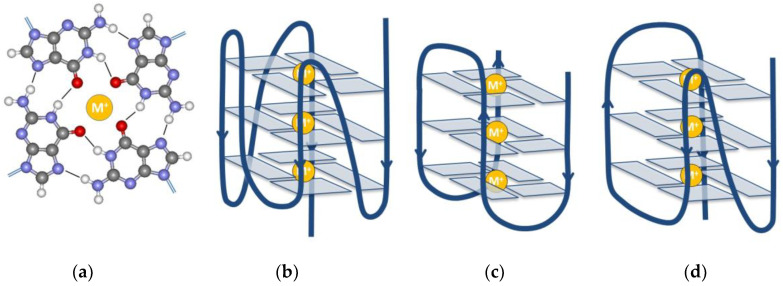
The main building block and the most frequently observed G-quadruplex structures. (**a**) Structure of a G-quartet with the metal ion (M^+^) in the middle of the plane. (**b**) Parallel; (**c**) antiparallel; (**d**) and hybrid structures.

The building blocks of the GQ are the G-quartets, which are planar structures, consisting of four guanine bases. The quartet is stabilized by Hoogsteen-type hydrogen bonds. Eight of such hydrogen bonds connect the four planar bases. In a GQ there are typically three of those G-quartets stacked on top of each other (Figure 1), although some GQs are formed only by two quartets, or very stable GQs can have four or more quartets. Cations are needed to stabilize the GQ form. Potassium is the main stabilizing ion, although sodium is also able to stabilize the structure. They are positioned in the middle of the GQ, either in the plane of the G-quartets or between them, depending on the size of the ion. Na^+^ is found in the middle of the quartets, while K^+^ fits only between the quartets because it has a higher ionic radius compared to sodium [20]. They stabilize the GQ by coordinating with the O_6_ atoms of the adjacent G-tetrad planes.

A GQ can adopt various forms according to the relative direction of the strands and to the form of the loops [2]. Figure 1 shows the mainly occurring forms. According to the strand orientation, they can be parallel (Figure 1b), antiparallel (Figure 1c) or hybrid (Figure 1d). The loop type is called a propeller in the case depicted in Figure 1b. Figure 1c shows a chair or lateral loops. Further classification based on the relative orientation of bases is described in detail in a recent review [2].

G-quadruplexes can be intramolecular (monomeric) or intermolecular (multimeric), which are formed by one or more than one nucleic acid molecules, respectively [8]. We focus our discussion on the monomolecular GQ and iM forms. 

The formation of non-canonical structures depends not only on sequence but also on the surrounding environment [21]. The cell interior contains macromolecules in a high concentration, (up to 30–40%) which reduces the space available and also influences the diffusion of other molecules. This effect is called macromolecular crowding [22]. This has a pronounced effect on the stability of proteins, nucleic acids and on cellular processes [21,23,24,25,26]. 

## 3. The i-Motif

**Figure 2:**
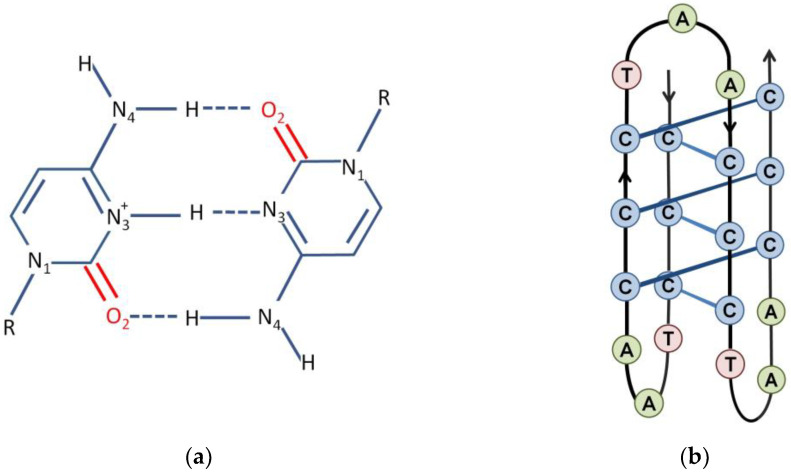
The structure of the i-motif. (**a**) A hemi-protonated cytosine-cytosine^+^ base pair is the main building block of the i-motif. (**b**) Schematic representation of the complete three-dimensional structure of an i-motif.

The complementary strand of the GQ-forming sequence is rich in cytosine bases. This can also form a four-stranded structure, called an i-motif (iM). This structure raised less attraction, since the formation of the i-motif was observed predominantly below a physiological pH [4,27,28]. The name comes from the intercalated nature of this form (Figure 2). The main building block of the i-motif is a hydrogen-bonded base pair formed by a neutral and a protonated cytosine. These bind through three hydrogen bonds (Figure 2a). According to theoretical calculations, the binding of this CC base pair is even stronger than the one between the CG pair [29]. I-motif consists of four cytosine strands in which two parallel duplexes are antiparallelly interspersed (Figure 2b). The formation of iM needs a pH where one of the cytosines is protonated on N_3_ and consequently, the iM is stable only under acidic conditions and its stability changes considerably in the range of pH = 4–7.

It has recently been demonstrated that the in vivo formation of iM is cell-cycle-dependent. Furthermore, iM structures were found in regulatory regions of the human genome, including promoters and telomeric regions. This fact means that they play regulatory roles in the genome [6].

Although iM folds only at an acidic pH, it was suggested to have an important role in the primordial RNA world [30]. They have also been suggested as promising targets for therapeutics use [31,32]. Recent studies indicate that the appearance of a folded iM is promoted by appropriate environmental conditions, even at a physiological pH.

## 4. High Pressure

Pressure studies are less common compared to the experiments probing the molecular stability by increasing the temperature or concentration of the destabilizing chemical agent. Although the pressure is an equivalently as important thermodynamic parameter as the temperature, its use is considerably hindered by technical difficulties. For biomolecules, the relevant pressure range is 0–1 GPa, which is 10,000 times higher than the atmospheric pressure and can be reached only by special experimental techniques [33,34]. The reason to perform pressure studies in spite of these difficulties is that the specific molecular information cannot be obtained by other techniques [35,36,37]. Since pressure and volume are conjugated thermodynamic parameters, experiments performed at different pressure values can provide volumetric parameters of the system. This way, volume differences (e.g., between folded and unfolded stets) and activation volumes can be obtained [38].

A large part of the biosphere is exposed to elevated pressure [39,40]. Hydrostatic pressure of roughly 10 m of water column is equivalent to the atmospheric pressure. In the deepest point of the ocean, one can experience more than 1.1 kbar (110 MPa) of pressure. Living organisms were observed in the deep sea, some of them were only barotolerant, but others were even barophilic organisms [41,42,43]. An investigation of the extreme environment has relevance in marine research and also in astrobiology [44,45].

According to the Le Chatelier–Brown principle, by changing the conditions of a thermodynamic system, the system changes on the way to counteract the change to reestablish a new equilibrium. In case of pressure, the equilibrium shifts in order to reduce the volume. If a molecule has more than one possible conformation, the one with lower volume is preferred under pressure. This has been observed in several macromolecular systems including proteins, lipid bilayers and also for GQs. It must be noted that the volume of the molecule in a solution includes the change in the solvent volume due to the interaction with the solute molecules (e.g., the hydration layer can have a different density compared to the bulk).

In the case of GQ-forming oligos, we must consider the folded (GQ) and unfolded single-stranded DNA (ssDNA) states of an oligo. Assuming the equilibrium reaction of GQ ↔ ssDNA, we can write:

(1) $${\left(\frac{\partial \ln K}{\partial p}\right)}_{T}=-\frac{\Delta V}{RT}$$

where *K* is the ratio of the relative abundances of the unfolded single-stranded DNA and folded GQ states:

(2) $$K=\frac{w_{ssDNA}}{w_{GQ}}$$

Δ*V* is the volume increase during the unfolding.

(3) $$\Delta V=V_{\mathrm{unfolded}}-V_{\mathrm{folded}}$$

A positive Δ*V* means that the unfolded state has a higher volume. As a consequence, the pressure stabilizes the folded state. In the case of a negative Δ*V* pressure, induced unfolding can be observed. It has to be noted that not all the publications use this notation. In some of them, Δ*V* is the excess volume of the folded state compared to the unfolded, which results in an opposite sign of the Δ*V* value. One has to look at the definition of Δ*V* carefully in order to interpret the results correctly. In this review, we present the Δ*V* values according to Eq. 3. As mentioned before, the volume of the molecule contains several terms, including the volume change in the solvent during hydration:

(4) $$V=V_{\mathrm{atom}}+V_{\mathrm{void}}+V_{\mathrm{thermal}}+\Delta V_{\mathrm{hydration}}$$

**Figure 3:**
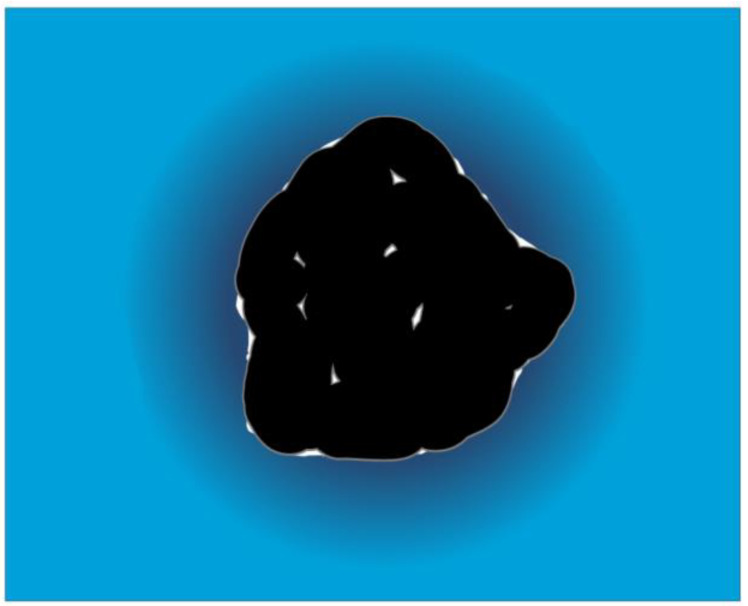
Volume contributions of a folded macromolecule. Black is the volume of the atoms; the cavities are shown in white. The thermal volume is gray. Dark blue indicates the hydration shell, which has higher density compared to the bulk water (light blue).

*V*_atom_ is the volume of the atoms as given by hard spheres. It is also called an intrinsic volume. For *V*_void_, small cavities are considered, which cannot accommodate any water molecules. These occur typically in the folded version of the macromolecules, while they are practically absent in a random chain form [36]. The sum of these two terms is sometimes called a molecular volume [46]. Thermal motions occupy a certain space called *V*_thermal_. This is connected to the thermal expansibility. The solvent around a solute molecule is denser than in the bulk phase, especially if the solute molecule contains charges at the surface. This density difference will result in a volume reduction caused by the solvent. This contribution to the effective volume of the solute molecule is given by Δ*V*_hydration_. It is also called an interaction volume since it is the result of the interaction of the solute molecule with the solvent. All these volume contributions are visualized in Figure 3.

Pressure effects several biomolecules including proteins, lipid bilayers and nucleic acids that have been studied extensively [35]. Pressure is known to act on the intermolecular interactions as well, for instance, inducing the dissociation of protein oligomers and some of the aggregates [47]. In this review we focus on pressure studies involving nucleic acids, especially in their noncanonical forms.

It must be emphasized that the pressure studies reviewed here are in vitro experiments, using pressure values that exceed even the highest pressure of the biosphere. Other sources of intracellular or intranuclear pressure, such as the osmotic pressure, are much smaller in magnitude. Although osmolites do have an effect on the macromolecules, these are out of scope of the present review. Such effects, especially those of the of osmolites, were extensively studied in professor Winter’s laboratory [40].

## 5. Experimental Methods to Study the Phase Transitions of Nucleic Acids under Pressure

Most experimental studies are based on optical spectroscopic methods such as UV-VIS absorption, IR absorption or fluorescence spectroscopy. The traditional method to study the “melting” of DNA (separation of the double helix into two single strands) is to follow the 260 nm absorption band which shows increased absorption upon denaturation (hyperchromic effect). The characteristic band for GQ formation is observable at the slope of this band at 295 nm [48]. This overlap causes the main difficulty in the measurement. Infrared spectroscopy, however, is sensitive to the molecular vibrations [49,50]. Conformation-sensitive infrared spectral bands can be used to follow the formation of the double helix, GQ or i-motif [28,50,51]. Fluorescence spectroscopy is also widely used in nucleic acid research [52]. Among the fluorescence techniques, the Förster resonance energy transfer (FRET) spectroscopy is the most often used. It has been successfully used for detecting conformational changes in small molecules, such as nucleic acid oligos [53,54]. FRET is sensitive to the distance of the two chromophores, which are usually attached to the two terminal positions of the oligo.

All these methods require optical access to the sample during the pressurization. There are principally two methods of performing optical spectroscopy under pressure: the diamond anvil cell and the optical cell based on the thick wall cylinder. The first has expensive diamond windows, which limit the sample size to a few times ten nanoliter, while the latter usually has sapphire windows, which can only withstand a few hundred MPa. These methods are described in detail in several reviews [33].

It has to be mentioned, that there is a remarkable technical difficulty in these studies, namely the determination of the pH during the experiment. In the case of temperature experiments, one usually chooses a phosphate buffer since it is relatively temperature insensitive. On the other hand, it is very pressure sensitive, 300 MPa increase can cause roughly one pH unit shift [55]. The Tris buffer is insensitive to pressure, but it changes its pH by 0.03 pH units/°C [56]. This means one must correct the pH shift caused by a pressure or temperature increase. The best way is to make a 3D fit for the obtained *T*_m_ values, as it was performed by Somkuti et al. [14]. They fitted the *T*_m_ as the function of pressure and pH, and the ∂*T*_m_/∂p|_pH_ value was obtained from the fitted function. Fortunately, the GQs are not very pH-sensitive, but the i-motifs are very pH sensitive [28,57,58].

## 6. Nucleic Acids and Pressure

Nucleic acids in their double helical form are relatively pressure insensitive. The double helical form of DNA was slightly stabilized by pressure, but contrary to proteins, no elliptic phase boundary was observed [59]. This is due to the small volume change during the melting of the double helix.

As intermolecular interactions are sensitive to pressure, it is not surprising that pressure was found to induce dissociation of the ribosome, the effect of which correlated with the cell death [60].

Due to the pressure insensitivity of double helices, the study of their pressure was not an attractive research field for a long time. Recently however, four-stranded tetraplex structures turned out to be pressure tunable. They gained an increased research interest in the high-pressure community since their pressure sensitivity allows for their volumetric characterization and the pressure tuning of their three-dimensional form.

## 7. GQ and Pressure

The pressure behavior of several GQ structures has been investigated [14,26,46,51,61,62,63,64,65,66]. The studied sequences are listed in Table 1, while the main results are summarized in Table 2.

The thrombin binding aptamer (TBA) d[G_2_T_2_G_2_TGTG_2_T_2_G_2_] is an antiparallel G-quadruplex that inhibits the activity of human alpha-thrombin. It binds to the human thrombin with high selectivity and an affinity that results in the inhibition of the fibrin clot formation [26,68,69]. The TBA is a two-quartet GQ in contrary to most of the GQs, which are three-quartet ones. This explains the relatively low temperature stability of the TBA. A systematic characterization of the TBA was performed in Sugimoto’s laboratory. They studied the effect of cations, pressure, crowding condition and pressure [26,69]. A volume change of Δ*V* = −55 cm^3^/mol was measured in the presence of 100 mM of K^+^ ion. A remarkable result of this study is that the pressure dependence of temperature stability and consequently the unfolding volume change was also considerably reduced under crowding conditions, i.e., in the presence of polyethylene glycol (PEG4000) [26].

The telomere region contains repeats of GGGTTA. At least three and a half parts of this repeat (i.e., the oligo with the d[(G_3_T_2_A)_3_G_3_] sequence containing 21 bases) is needed to form a GQ structure. Several variants of this oligo were studied. Htel22 (also called a tel22 or simply a Htel) contains an additional adenine base at the beginning, while Htel26 (or tel26) contains three additional adenines at the beginning and two of them at the end of the sequence. The latter does not conform completely with the telomere sequence, which does not contain any AAA repeat.

Htel variants were studied widely, but regarding high pressure, there are only a few experimental works. Macgregor’s laboratory studied the pressure behavior of the Na+ stabilized Htel22 oligo d[A(G_3_T_2_A)_3_G_3_] [46]. Their experiments show the stabilization of the GQ form upon an increasing concentration of Na^+^ in the range of 20–100mM. The midpoint of the unfolding transition (T_m_) increased by c.a. 15 °C (from 39.6 °C to 54.9 °C) in this concentration range. Similar stabilizing effects have been observed by Molnár et al., in the case of another GQ originating from the genome of the hepatitis B virus [13]. Parallel to the temperature stabilization of Htel22, the magnitude of the unfolding volume change decreased from 67 cm^3^/mol to 56 cm^3^/mol, as a result of the Na^+^ concentration increase from 20 mM to 100 mM [46]. The hydration of one of the sodium ions released from the middle of the GQ can explain 5.9 cm^3^/mol, which means that the release of three Na ions is responsible for the c.a. 18 cm^3^/mol volume change and the rest comes from the interaction volume caused by the increased surface of the unfolded oligo and of the disappearance of the buried void volume. The authors performed a very detailed theoretical calculation regarding the origin of the volume change, which indicated a delicate balance of the changes in the different contributions to the volume (see Eq. 3). It must be mentioned that different experimental methods provided slightly different *T*_m_ and Δ*V* values in this study. This might be the result of the different oligo concentrations used in these techniques.

Li et al. obtained slightly smaller Δ*V* values for the same oligo stabilized by a Na^+^ ion [66]. This discrepancy can probably be explained by the fact that they calculated the Δ*V* values at 57 °C and their pH was also slightly higher. This draws attention to the fact that GQ structures are very sensitive to environmental conditions, and experimentalists should compare results only if the chemical–physical conditions were exactly the same. This paper also investigates the effect of mutations on the loop regions which will be discussed later.

The self-crowding effect of Htel was investigated by Somkuti et al. [51]. Using florescence (FRET) and infrared-absorption spectroscopic techniques, they obtained different pressure stabilizing profiles, since these techniques require considerably different concentrations of the oligos. Micromolar concentration (10^−5^ M = 0.08 mg/mL) in the FRET experiments showed a negative unfolding volume, as it was obtained by earlier experiment of other groups too. In the concentration range needed for the infrared experiments (2–75 mg/mL), the trend changed, and the ∂p/∂*T*_m_ value became positive. One possible explanation is that the crowded environment might favor the parallel conformation of Htel instead of the hybrid conformation, which is present at low concentrations [70]. This effect is also known from earlier studies on the Htel structure, where discrepancies of crystallographic and NMR studies could be resolved only by assuming a conformational change, while the concentration increased in the crystallization process [71]. Aggregation of the GQs at a high concentration is the other possible explanation for the change in the slope of the phase transition line in the T-p phase diagram. However, it is known from the high-pressure protein experiments that aggregates are very pressure sensitive, even a small pressure can dissociate them [72,73]. However, in the case of Htel, the high concentration form is more pressure stable; pressure stabilizes the high concentration form which makes this explanation implausible.

The pressure behaviors of three human oligos were measured by Molnár et al. [62]. The c-MYC, KIT and VEGF oligos have different lengths and sequences, but all of them have a predominantly parallel structure. KIT was, however, reported to have a unique structure containing four loops instead of the usual three [74]. The unfolding volumes of all these GQs were negative. The pressure dependence of *T*_m_ was considerably smaller than in the case of other oligos investigated before. The magnitudes of the volume changes are in the range of the volume of a water molecule or less. All the phase boundaries of the p-T diagram were linear, without any curvature. This indicates that the second derivatives of the thermodynamic potentials did not change considerably at the unfolding temperature.

Liu et al. studied the same c-MYC sequence [64]. They predicted an elliptic phase boundary for their c-MYC variant, similar to that which was found in the case of several proteins [33,35,72]. The elliptic boundary was calculated from the thermodynamic parameters obtained at an atmospheric pressure or within a relatively low (≤160 MPa) pressure range. This means that only a small part of the predicted boundary was experimentally accessed. 

This work seems to be contradictory to the previously mentioned experimental work, where c-MYC was measured up to 600 MPa and not an elliptic but a rather linear phase boundary was found. However, one has to mention that Liu et al. used quite a low K^+^ ion concentration of 1mM and 0.1 mM, which is known to influence the stability of the GQ structure considerably [13]. Consequently, we must emphasize here again the importance of the sensitivity of the GQ to small environmental changes.

Besides the human genome, there are plenty of potentially GQ-forming sequences in the viral genomes as well [16,17]. Lavezzo et al. [10] searched through the genomes of several viruses to find such sequences. In the case of the hepatitis B virus, the highest G-score was obtained for the GGC TGG GGC TTG GTC ATG GGC CAT CAG (NC_003977.2:1204..1230 (+strand)) sequence. This can be found in the coding region of the polymerase protein. Other potentially GQ-forming sequences with slightly lower scores were also identified in this genome. Biswas et al. [75] found a conserved sequence (GGG AGT GGG AGC ATT CGG GCC AGG) in the promoter region of the envelope-coding gene of the S protein of the virus. Another promising GQ-prone sequence is the GGG TGG CTT TGG GGC ATG G (NC_003977.2 NC_003977.2:1886..1904 (+strand)), which can be found in the C protein’s signaling region of the virus.

**Figure 4:**
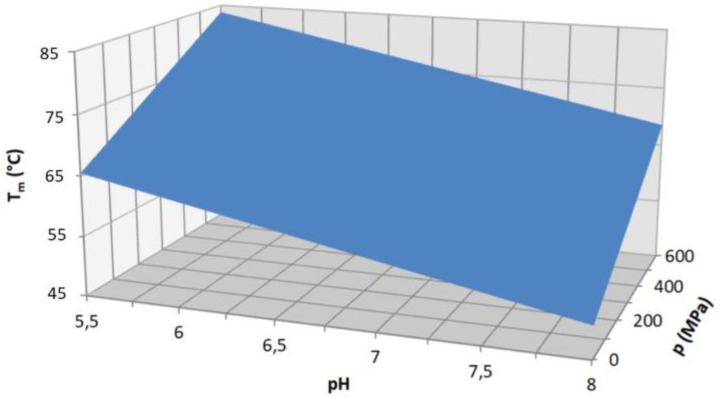
Three-dimensional multivariate fit of the transition temperature vs. pH and pressure, in case of the HepB1 oligo measured by Somkuti et al. [14].

These oligos named HepB1-3 were investigated in our laboratory [13,14] (HepB1: GGC TGG GGC TTG GTC ATG GGC CAT CAG, HepB2: GGG AGT GGG AGC ATT CGG GCC AGG G HepB3: TTG GGT GGC TTT GGG GCA TGG AC). All three of the oligos were proven to be able to form GQ experimentally [13,14]. Their pressure behavior was investigated. These oligonucleotides turned out to be pH sensitive, which complicated the evaluation of the data. The authors performed a three-dimensional fit which provided the *T*_m_(*p*, pH) function. (Figure 4) *T*_m_ was assumed to be a linear function of both *p* and the pH. The ∂*T*_m_/∂*p* value was determined by the fit, and the Δ*H* obtained from the individual measurements allowed calculating the Δ*V* from the Clausius–Clapeyron equation. The Δ*V* values were small for HepB1 around 17 cm^3^/mol, while for the other two oligos, Δ*V* was around zero [14]. Additionally, the authors showed that these viral GQs can be stabilized by those ligands that were developed for the tumor therapy (i.e., for stabilization of human GQs, such as Htel.) All of the studied HepB oligos could be stabilized by these ligands (TMPyP4, BRACO19 and PhenDC3). This might have an impact on the fight against viral infections. 

The binding of berberine to an RNA GQ of the SARS-CoV-2 virus was investigated in Winter’s laboratory [18]. Berberine has antiviral potential, and it is a possible therapeutic candidate against COVID-19. They observed a negative binding volume of −13 cm^3^/mol. This together with the negative entropy change observed indicated the decrease in packing defects (void volume) upon ligand binding. The berberine-bound structure turned out to be more compact and less flexible.

The effect of the loop length on the stability of GQs was systematically studied by Guedin et al. [76]. Unfortunately, these authors did not perform any pressure studies; however, their results on the heat stability are still remarkable. They concluded that long loops destabilize the quadruplexes by 2 °C/nucleotide in the case of K^+^, while this effect is smaller (1.5 °C/nucleotide) in presence of Na^+^. One has to mention that the loop length can also influence the topology, which also influences the stability. The second important conclusion is that GQs can be produced even in the case of long loops, so the 7-nucleotide loop length limit used in bioinformatic algorithms is arbitrary and too stringent. This limit might lead to overlooking GQ structures. Nowadays, a number of GQs have been discovered with a long loop, forming G-quadruplex-duplex hybrid forms [77]. As mentioned, only the temperature stability was investigated as a function of the loop length. The author is unaware of any pressure studies of GQs with different loop sizes, but for the full understanding of the volumetric contributions of the loop regions, we will definitely need studies of the effect of the loop length on the pressure stability.

The loop sequence also influences the stability of GQs. Sugimoto’s group investigated the TBA sequence by varying the loop region [78]. They investigated the effect of loop alteration on the stability and volumetric properties of TBA. Mutations were introduced in the first and second loop: T3A and G8T. In separate experiments, these loops were replaced by a hydrocarbon chain containing a C12 linker (spacer C12 CE). The stability decreased in all the cases, but the volume difference increased, except for G8T. Although the C12 linker eliminates the stacking of the loops to the GQ core structure, this linker should interact with the structure in a way so that the volume of the folded structure increases.

The loop region of Htel was varied by Li et al. [66]. They found the destabilization of the GQ structure in almost all the cases except for two variants with mutation in the middle loop, but only when they were stabilized by Na^+^. Interestingly, in these cases the magnitude and volume change was considerably lower, compared to the original Htel sequence.

The role of the loops in the volumetric properties of GQs was further emphasized by a recent study [65]. These authors compared the pressure effect on the stabilities of the tetramolecular d[TGGGGT]_4_ GQ and the monomolecular Pu12T13T GQ. Contrary to PuT12T13, the tetramolecular GQ did not show any significant pressure dependence up to 200 MPa. Both structures had parallel strand orientation, although TG4T GQ was stabilized by high concentrations of Na^+^, while PuT12T13 was formed in the presence of low concentrations of the K^+^ ion. Since tetramolecular GQ does not have any loops, the authors concluded that the Δ*V* values for the monomolecular GQs are due to the presence of the loop regions in those structures [65].

The effect of molecular crowding on the stability of the GQ structures was also studied using crowding agents, such as polyethylene glycol (PEG). The biological relevance of these studies is supported by the fact that nucleic acids may prefer non-canonical structures to canonical ones under cellular environments, such as molecular crowding and confined environments [21]. Sugimoto’s laboratory obtained a drastic decrease in the folding volume change (by a factor of four) of the TBA in the presence of ethylene glycol or by PEG molecules with either a molar mass of 200 or 4000 [26]. A similar decrease was found by the same group in the case of Htel when PEG200 was present [79]. 

High-pressure single-molecule FRET experiments by Knop et al. found that molecular crowding can counteract the unfolding effect of the pH and pressure in the case of Htel [80].

## 8. i-Motif under Pressure

Unfortunately, the diversity of the volumetric properties of different iM sequences is hardly studied; there are only a few high-pressure works on iM.

An early study on the pressure stability of iMs was performed by Takahashi et al. [57]. They investigated the artificial sequence d[CGG(CCT)_10_CGG]. They obtained a stabilization of the iM using pressure: *T*_m_ increased from 38.8 °C (atmospheric pressure) to 61.5 °C under 400 MPa pressure. However, iM is very sensitive to pH, roughly a 20 °C decrease in *T*_m_ at each pH unit increase was observed in the case of Htel-iM below pH6 [28]. The authors concluded that the observed stabilization can at least be partially associated with the pH shift of the phosphate buffer caused by the pressure and not with the direct effect of the pressure.

Liu et al. [81] studied the C-rich DNA sequence from the promoter region of the human c-MYC oncogene d[TTACCCACCCTACCCACCCTCA]. They did not find any significant volume change during the unfolding of the iM structure.

Lepper et al. [58] studied the d[CCC(TAACCC)_3_] sequence using NMR and CD spectroscopic methods. They pointed out the importance of the correction for the changing of pH under pressure. Such a correction even changed the sign of the Δ*V*. The Δ*V* turned out to be very pH-dependent, with a zero point at pH4.6.

Somkuti et al. studied the pressure sensitivity of the human telomeric iM, which formed the complementary strand of Htel GQ [28]. They used infrared and fluorescence (FRET) spectroscopies. Their data proves that the unfolding process is not a simple cooperative one but contains more unfolding steps. Infrared spectroscopy could distinguish between the breakage of the outer and inner hydrogen bonds. They have proven that the breakage of the outer hydrogen bonds precedes the complete unfolding of the structure. Careful correction for the pressure and temperature induced changes that led to a negative unfolding volume of 15 ± 4 cm^3^/mol. In this case, there is no stabilizing central ion, as in the case of GQs, which means that the whole volume change can be assigned to the hydration and cavity loss of the oligo. Interestingly, the FRET-labeled oligo shows a smaller DV value of 11 ± 2 cm^3^/mol. The discrepancy between the infrared and fluorescence experiments is still not completely understood but the binding of the chromophores to the oligonucleotide could also contribute to the reduction of the volume of the folded molecule. On the other hand, the IR measures the breakage of the internal hydrogen bonds directly, while the FTIR signal changes only if conformational changes happen there, due to the loss of hydrogen bonds that can stabilize the folded structure. An important conclusion of this study is that Htel-iM is destabilized by pressure.

Winter’s laboratory found an induction of the folded iM structure by adding oligomers of the intrinsically disordered protein α-synuclein [82]. Quite a low pressure of 1 kbar led to an almost complete disappearance of the folded iM fraction. This can be ascribed to the dissociation of the protein oligomers. Protein intermolecular binding is known to be very pressure sensitive [73,83]. The determined Δ*V* is also very close to the one obtained from the dissociation of α-synuclein oligomers [84], which indicates a tight connection between the binding and iM stability.

## 9. Conclusions

High-pressure studies on GQs and iMs are very important for understanding these structures and the role of the intracellular milieu that may cause stabilization or destabilization of these structures in vivo.

**Figure 5:**
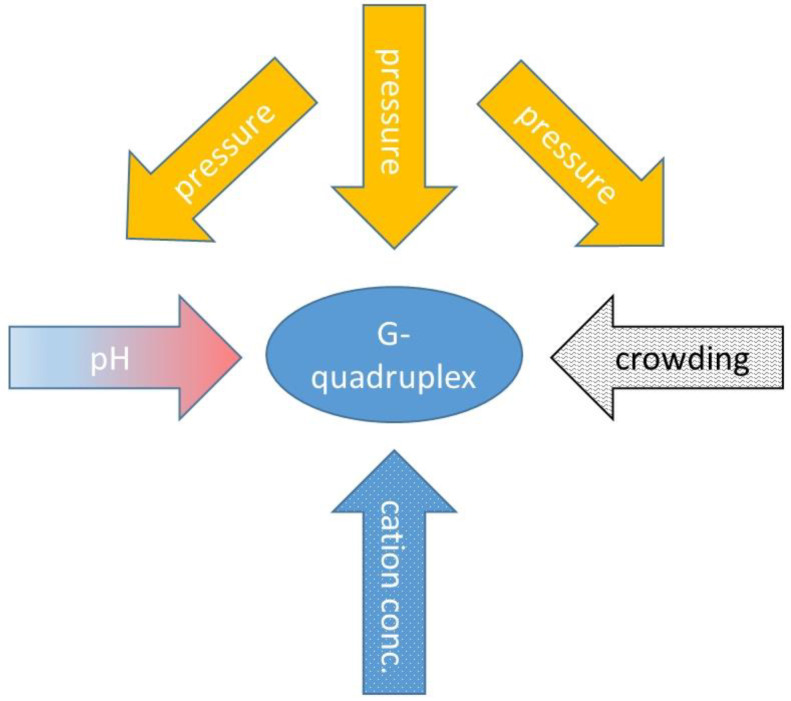
Complexity of the direct and indirect pressure effects in case of GQ.

Looking to the values in Table 2, it is obvious to see that the experimental conditions are very important in these measurements. Diversity of the data is partially the result of the different physico-chemical environments, and of the slight modifications of the oligonucleotides studied. Fan et al. used several experimental techniques (pressure perturbation calorimetry, densitometry and high-pressure UV-absorption spectroscopy) and obtained slightly different transition temperature values for the same oligomer [46]. Somkuti et al. used fluorescence and FTIR spectroscopic methods and they sowed slightly different transition points even if they were measured exactly on the same sample [51]. Differences in the cation concentration, pH and concentration of the oligos, can also influence the stability of the GQ structure. We can also observe that the FRET experiments show a significantly smaller pressure sensitivity, which is consequently lower than the Δ*V* values compared to the other techniques. This effect has to be investigated further systematically, to achieve a full understanding of this difference. Furthermore, crowding conditions can considerably influence the stability [26,51]. We can conclude that the stability of the GQ is very sensitive to environmental factors, and a comparison of different publications can be performed only with extra care, taking into account these factors and their interactions. Figure 5 illustrates this complexity in the case of GQs.

There is still a lot of work to be conducted in order to fully understand the volumetric aspects of GQs and iMs. Furthermore, a number of oligos have not been characterized from this point of view. Due to the COVID-19 pandemic, the GQ-prone sequences in the genome of the SARS-CoV-2 virus came to the focus, but their volumetric characterization is still not complete.

## Figures and Tables

**Table 1:** The DNA sequences of the oligos reviewed in this paper.

Name	Sequence
c-MYC	d[TGAGGGTGGGTAGGGTGGGTAA]
HepB1	d[GGC TGG GGC TTG GTC ATG GGC CAT CAG]
HepB2	d[GGG AGT GGG AGC ATT CGG GCC AGG G]
HepB3	d[TTG GGT GGC TTT GGG GCA TGG AC]
Htel22	d[AGGGTTAGGGTTAGGGTTAGGG]
Htel26	d[AAAGGGTTAGGGTTAGGGTTAGGGAA]
KIT	d[AGGGAGGGCGCTGGGAGGAGGG]
Pu22T12T13	d[CGGGGCGGGCCTTGGGCGGGGT]
TBA	d[GGTTGGTGTGGTTGG]
TG4T	d[TGGGGGT] tetramolecular
VEGF	d[TTGGGGCGGGCCGGGGGGCGGGGTT]
c-MYC iM	d[TTACCCACCCTACCCACCCTCA]
Htel-iM	d[CCCTAACCCTAACCCTAACCC]

**Table 2:** The unfolding volumes and temperatures of DNA GQs, and the corresponding experi-mental conditions.

Name	Cation	Cation Conc.	Δ*V* (cm^3^/mol)	*T*_m_ at 1bar (°C)	Oligomer Conc. (μM)	N	Topology	pH	Buffer	Ref
TBA	K^+^	100 mM	−54.6 ± 4.2	52.6 ± 3.4	40	15	AP	-	TRIS 30 mM	[26]
Htel22	Na^+^	100 mM	−38.4 ± 10.1	54.6 ± 0.9	20	22	AP	7.4	TRIS 10 mM	[66]
Htel22	Na^+^	2 0mM	−68 ± 2 ^1^	39.6 ± 0.7 ^1^	10–300 ^4^	22	AP	7.0	Na phosph 10mM	[46]
Htel22	Na^+^	50 mM	−60 ± 2 ^1^	47.9 ± 0.7 ^1^	10–300 ^4^	22	AP	7.0	Na phosph 10mM	[46]
Htel22	Na^+^	100 mM	−56 ± 2 ^1^	54.9 ± 0.6 ^1^	10–300 ^4^	22	AP	7.0	Na phosph 10mM	[46]
Htel22	K^+^	100 mM	−42.7 ± 6.7	64.6 ± 2.2	20	22	H	7.4	Tris 10 mM	[66]
c-MYC	K^+^	170 mM	−16.9 ± 1.8	83.4 ± 1.3	2	22	P	7.4	K-phosph 100 mM	[62]
KIT	K^+^	170 mM	−6.2 ± 0.9	58.5 ± 0.4	2	22	P	7.4	K-phosph 100 mM	[62]
VEGF	K^+^	170 mM	−18.1 ± 4.6	78.8 ± 1.1	2	24	P	7.4	K-phosph 100 mM	[62]
Htel26	K^+^	20 mM	−69 ± 7 ^2^	NA	300	26	H	7.0	tetrabutylammonium phosphate	[63]
c-MYC	K^+^	0.1 mM	−30 ± 4	59.1	25	22	P	7.0	Cs-phosp 10 mM	[67]
HepB1	K^+^	140 mM	17.8 ± 3.8	NA	1	27	NA	7.4 ^3^	K-phosp/TRIS	[14]
HepB1	K^+^	100 mM	16.1 ± 2.0	53.8	2400	27	NA	7.4	Bis-TRIS 100 mM	[14]
HepB2	K^+^	140 mM	−3.7 ± 0.6	51.9	1	25	NA	7.4 ^3^	K-phosp/TRIS	[14]
HepB3	K^+^	140 mM	2.0 ± 1.1	41.4	1	23	NA	7.4 ^3^	K-phosp/TRIS	[14]
TBA (+PEG)	K^+^	100 mM	−13.1 ± 1.0	60.6 ± 3.2	40	15	AP	7.0	TRIS 30 mM	[26]
Htel22	K^+^	170 mM	−19 ± 3	63	10	21	H	7.4	K-phosph 100 mM	[51]
Htel22	K^+^	200 mM	6.4 ± 1	NA	9400	21	P?	7.4	Bis-TRIS 200 mM	[51]
TG4T	Na^+^	1 M	1.5 ± 2.3	68.9	100	4 × 7	P	7.5	TRIS 10 mM	[65]
Pu22T12T13	K^+^	2 mM	−38 ± 10	71.8	100	22	P	7.0	tetrabutylammonium phosphate	[65]

^1^ Weighted average of the published values obtained from different experimental techniques. ^2^ K+ induced volume change at 25 °C. ^3^ Ph7.4 K-phosphate buffer was used for determination of the *T*_m_ at atmospheric pressure, the pH dependence was ruled out by a 3D fitting. ^4^ Depending on the used experimental techniques.

## Data Availability

Not applicable.
